# Abdominal Wall Closure in Elective Midline Laparotomy: The Current Recommendations

**DOI:** 10.3389/fsurg.2018.00034

**Published:** 2018-05-23

**Authors:** René H. Fortelny

**Affiliations:** ^1^Department of General, Viszeral and Oncologic Surgery, Wilhelminenspital, Vienna, Austria; ^2^Medical Faculty, Sigmund Freud University Vienna, Vienna, Austria

**Keywords:** midline closure, small bite technique, prophylactic mesh, incisional hernia, prevention

## Abstract

**Introduction:**

The risk of developing an incisional hernia after primary elective median laparotomy is reported in the literature as being between 5 and 20 percent. The goal of this systematic review was to evaluate different closure techniques for midline laparotomies and the use of additional prophylactic mesh augmentation for midline closure in high risk patients.

**Method:**

A systematic literature search was performed until September 2017. The quality of the RCTs was evaluated and analysed. The data are reported in accordance with the Reporting Items for Systematic Reviews and Meta-Analyses (PRISMA) statement.

**Results:**

In the systematic review for closure techniques a total of 23 RCTs and 9 RCTs for the use of prophylactic mesh were included. In elective midline closure the use of a slowly absorbable suture material for continuous closure using the small bites technique results in significantly less incisional hernias than a large bites technique (OR 0.41; 95% CI 0.19, 0.86). The use of prophylactic mesh versus the suture closure of the midline achieved a significant reduction of the incisional hernia rate [OR 0.14 (95% CI 0.07–0.27)].

**Conclusions:**

Based on the currently evidence in midline closure after elective laparotomy in the small bites technique can be recommended to reduce significantly the rate of incisional hernia. The additional use of a prophylactic mesh in high risk patients can significantly reduce the occurrence of incisional hernia.

## Introduction

After primary elective median laparotomy the risk of developing an incisional hernia is found in the published studies to be between 5 and 20 percent (1). One of the crucial risk factors of the genesis of incisional hernias is the malfunction of collagen synthesis. A direct correlation between the formation of a reduced collagen 1/3 quotient and the development of an unstable scar was detected by Friedman in 1993 (2). Other main risk factors are found to be obesity, steroid therapy, malnutrition, nicotine abuse, and other connective tissue diseases.

But what about further risk factors associated with surgical performance like closure technique, suture material, surgical experience? In face of the paradigm shift in hernia surgery triggered by Usher (3) with the first use of prosthetic meshes at the beginning of the 1980s the problem of appropriate and safe closure after midline laparotomy is still an issue.

Over several generations of surgeons, the mass-layer single stitch suture has been the most commonly used technique for closure of the midline incision. Currently, this technique has had varying degrees of success in its application, especially in difficult secondary closures, e.g., burst abdomen complications. Parallel to the developments in meshes, there are actually different suture materials applicable for the closure of the abdominal wall in incisional hernia surgery as well as for primary midline closure.

In 1993 Leif Israelsson and his Swedish author group found a superiority of the continuous closure technique with a defined suture/wound length ratio of at least 4:1 (4, 5). The discussions regarding the ideal suture whether it should be “non - or slowly absorbable” took quite a long time. The evidence-based data for the ideal primary abdominal closure on the basis of numerous studies and meta-analysis (6–9) seems to have been adequately established. The most recent meta-analysis of primary laparotomy closure by Diener et al. (9) considered the continuous suture technique with a suture/wound length ratio of at least 4:1 using a monofilament, slowly absorbable suture to be the state of the art.

Nevertheless, controversial results in controlled trials prompt further studies and the discussion of essential issues. The interpretation of the surprising results of the INSECT study (10), contrary to general evidence, raised the suspicion that there could be some bias present, mainly with regard to surgical accuracy.

## Methods

The aim of the systematic review was to evaluate published RCTs comparing techniques for fascial closure of a laparotomy with the primary endpoint of incisional hernia. The second aim was to evaluate the addition of a prophylactic, non-absorbable synthetic mesh compared to primary midline closure using only sutures. In the first systematic reviews only randomized controlled studies (Level of evidence 1b) and in the second also comparative studies (Level of evidence 2b) with at least 12 months of follow up were included. A systematic search was performed in the the databases of Medline, EMBASE, Cochrane, SCOPUS and CINAHL including all published paper till September 2017.

## Results

The literature search for the fascia closure techniques obtained the inclusion of 23 RCTs (Table 1). Six studies described the suture to wound length ratio of 4:1 but only the study of Millbourn et al (11) and the STITCH –trial (12) analysed the ratio. So in fact these two studies are the only available RCT’s concerning the defined type of closure with slowly absorbable monofilament suture material in continuous technique comparing small versus large bite technique which is supposed to be the recommended closure technique after elective midline laparotomy regarding the occurrence of incisional hernia in the recent published analysis of Henriksen et al (13) (OR 0.41; 95% CI 0.19, 0.86; Table 2). The discussion whether interrupted or continuous closure techniques are recommended seems to be solved after the metaanalysis of Diener et al (9) which was significantly in favor of the continuous technique. In the contrary in the last MATCH-Metaanalysis (13) showed no significant difference found between these two techniques [OR 1.20 95% CI (0.84, 1.71) *p* = 0.31]. The comparison of non-absorbable and fast-absorbable sutures obtained no significant differences on incisional hernia rate [OR 0.89, 95% CI (0.69, 1.15) *p* = 0.38].

**Table 1:** Overview of 23 RCT's regarding midline closure.

**Study**	**Number of patients(group A/group B)**	**Type of laparotomy**	**Intervention**	**Comparison**	**SL/WL Ratio**	**Follow-up(months)**	**Outcome measure**
Agrawal 2009	147(40/47/45/42)	Emergency midline	Non-absorbable continuous mass-closure (polypropylene 1–0)Non-absorbable interrupted mass-closure (polypropylene 1–0)	Fast-absorbable continuous mass-closure (polyglactin 1–0)Fast-absorbable interrupted mass-closure (polyglactin 1–0)	4:1	48	Incisional hernia SSI, dehiscence, suture sinus
Berretta 2010	191(63/63/65)	Elective midline	Non-absorbable monofilament mass-closure (Premilene 1–0)Non-absorbable multifilament interrupted fascial closure (Ethibond 2–0)	Slowly-absorbable monofilament mass-closure (PDS 1–0)	4:1	36	Incisional hernia,SSI, dehiscence, scar pain
Bloemen 2011	456(233/233)	Elective and emergency midline	Non-absorbable monofilament(Prolene 1–0)	Slowly-absorbable monofilament(PDS 1–0)	4:1	35	Incisional herniaSSI and suture sinus
Bresler 1995	235(70/71/62)	Elective midline	Slowly absorbable PDS continuous suture(1 or 2)	Fast absorbable, continuous suture polyglactine	N/A	12	Incisional hernias, SSI, dehiscence, suture sinus
Cameron 1987	284(143/141)	Elective and emergency midline	Non-absorbable monofilament interrupted mass closure (Prolene 1–0)	Slowly-absorbable monofilament interrupted mass closure(PDS 1–0)	N/A	15	Incisional hernia, SSI, dehiscence, palpable knots, pain, suture sinus
Carlson 1995	225(112/113)	Elective and emergency midline	Non-absorbable monofilament nylon continuous mass closure (Ethilon 0–0 loop)	Slowly absorbable monofilament polygluconate nylon continuous mass closure (Maxon 0–0 loop)	N/A	24	SSI, wound dehiscence and incisional hernia
Colombo 1997	614(308/306)	Elective midline	Slowly-absorbable monofilament polygluconate continuous (Maxon 1–0)	Fast-absorbable multifilament polyglucolic acid, Interrupted (Dexon 1–0)	N/A	33	Incisional hernia, dehiscence
Corman 1981	161(49/53/59)	Elective and emergency midline	Monofilament non-absorbable polypropylene, interrupted single layer (Prolene)Multifilament non-absorbable uncoated nylon, interrupted single layer(Nurolon)	Fast absorbable, multifilament single layer (Vicryl)	N/A	19	Incisional hernia, SSI, dehiscence, suture sinus
Deerenberg 2015	560(284/276)	Elective midline	Slowly-absorbable,monofilament, continuous single layer, small bites (PDS 2–0)	Slowly-absorbable,monofilament, continuous mass closure, big bites (PDS 1–0 loop)	4:1, 5:1	12	Incisional hernia, SSI, burst abdomen
Deitel 1990	84(42/42)	Elective midline	Slowly-absorbable, monofilament polygluconate, continuous single layer (Maxon 1–0)	Fast-absorbable, multifilament polyglucolic acid, continuous single layer (Dexon 1–0)	N/A	24	Incisional hernia, seroma, SSI
Donaldson 1982	231(80/74/77)	Elective and emergency paramedian	Continuous polypropylene (Prolene 1–0)	Continuous chromic catgut 1–0, Continuous polyglycolic acid (Dexon 1–0)	N/A	12	Incisional hernia, SSI, dehiscence, suture sinus
Gislason 1995	599(203/199/197)	Elective and emergency, subcostal, transverse and midline	Continuous mass polyglucanate (Maxon loop)Continuous mass polyglactin(Vicryl)	Interrupted polyglactin(Vicryl)	N/A	12	Incisional hernia, burst abdomen
Gurjar 2012	200(100/100)	Elective and emergency midline	Continuous polypropylene, intermittent Aberdeen knot at every 4 stitch (Prolene 1–0)	Interrupted simple stitch polypropylene(Prolene 1–0)	N(A	12	Incisional hernia, SSI, dehiscence,
Gys 1989	132(67/65)	Elective and emergency, subcostal and midline	Non-absorbable polyamide continuous layered closure(Ethilon 1–0)	Slowly-absorbable monofilament polygluconate continuous layered closure with (Maxon 1–0)	N/A	12	Incisional hernia, SSI, burst abdomen
Hsiao 2000	340(184/156)	Elective laparotomy, midline, subcostal, paramedian and transverse	Slowly-absorbable monofilament polydioxane continuous layered closure(PDS loop 0–0)	Absorbable multifilament polyglactin continuous layered closure with(Vicryl 0–0)	N/A	24	Incisional hernia, SSI
Krukowski 1987	757(374/383)	Elective and emergency midline	Non-absorbable monofilament continuous may closure(polypropylene)	Slowly absorbable monofilament continuous mass closure (polydioxane)	N/A	12	Incisional hernia, SSI, pain
Lewis 1989	200(105/95)	Elective and emergency midline	Non-absorbale polypropylene continuous layered closure(Prolene 1–0)	Fast absorbable polyglycolic acid Interrupted Smead Jones (Dexon 1–0)	N/A	60	Incisional hernia, SSI, dehiscence,
Millbourn 2009	737(381/356)	Emergency or elective midline	Slowly absorbable polydioxane, continuous single layer, small bites (PDS 2–0)	Slowly absorbable polydioxane, continuous single layer, big bites (PDS 1–0)	4:1, 5:1	12	Incisional hernia, SSI, dehiscence,
Osther 1995	204(100/104)	Emergency or elective paramedian, transverse and oblique	Slowly absorbable polygluconate,Single layer interrupted (Maxon 0–0)	Fast-absorbable polyglycolic acid, single layer interrupted (Dexon 0–0)	N/A	12	Incisional hernia, SSI, dehiscence
Richards 1983	571(286/285)	Emergency or elective paramedian, transverse and oblique	Non-absorbable polypropylene, continuous layered(Prolene 0–0)	Fast-absorbable polyglycolic acid, layered Interrupted Smead- Jones (Dexon 0–0)	N/A	12	Incisional hernia, SSI, dehiscence, hematoma
Sahlin 1993	988(345/339)	Emergency or elective, paramedian, transverse, midline	Slowly-absorbable monofilament polygluconate, continuous (Maxon 0–0)	Fast-absorbable multifilament polyglactin, Interrupted(Vicryl 0–0)	N/A	12	Incisional hernia, SSI, dehiscence
Seiler 2009	625(210/205/210)	Elective midline	Slowly absorbable monofilament, polydioxanone, continuous (MonoPlus)Slowly absorbable polydioxane continuous (PDS)	Fast-absorbable multifilament polyglactin, interrupted Vicryl	4:1	12	Incisional hernia, SSI, burst abdomen
Wissing 1987	1539(365/379/370/377)	Ermergency and elective midline	Slowly absorbable polydioxane, continuous single layer(PDS 0–0)Non-absorbable, continuous single layer (nylon 1–0)	Fast-absorbable multifilament polyglactin, interrupted(Vicryl 1–0)Fast-absorbable multifilament polyglactin, continuous(Vicryl 1–0)	N/A	12	Incisional hernia, SSI, dehiscence, suture sinus, pain,

**Table 2:** Analysis of 2 RCT's of Meta analysis of Henriksen.

	**Suture techniqueSmall bites**		**Suture techniqueBig bites**		**Odds Ratio**	
**Study**	Incisional hernia	Total Number	Incisional hernia	Total Number	Weight	M-H, Random, 95% CI
Millbourn 2009	14	250	49	272	46.1%	0.27 (0.15, 0.50)
Deerenberg 2015	35	268	57	277	53.9%	0.58 (0.37, 0.92)
Total (95% CI)		518		549	100%	0.41 (0.19, 0.86)
Total events	49		106			

Heterogeneity: Tau^2^ = 0.22; Chi^2^ = 3.77, df = 1 (*p* = 0.05); I^2^ = 73%.

Test for overal effect: Z = 2.35 (*p* = 0.02).

9 RCTs were selected in the search for the use of prophylactic mesh in midline closure after elective laparotomy in high risk patients (Table 3). The analsysis of Payne et al (14) including 8 RCT (Table 4) yielded significant reduction of the incisional hernia rate (OR 0.14 (95% CI 0.07–0.27) comparing the use of prophylactic mesh versus the suture closure of the midline in high risk patients. Another study in this field was recently published by Jairam et al (15) The study included patients aged 18 years or older, who were treated by elective midline laparotomy and had either an abdominal aortic aneurysm or a body-mass index (BMI) of 27 kg/m^2^ or higher. The study patients were randomly assigned to one of three treatment groups: primary suture; onlay mesh reinforcement; or sublay mesh reinforcement. The primary endpoint was defined by the incidence of incisional hernia in a 2 years follow-up. In total 480 patients were included in the primary analysis: 107 in the primary suture only group, 188 in the onlay mesh reinforcement group, and 185 in the sublay mesh reinforcement group. After one year in total 92 patients were identified with an incisional hernia. The rate of incisional hernias was 30% in the group of primary suture, 13% in the group of onlay mesh reinforcement and 18% in the group of sublay mesh reinforcement. The detailed analysis of the hernia rate in comparison between the different groups detected: onlay mesh reinforcement vs primary suture an Odds Ratio of 0·37 (95% CI 0·20–0·69; *p* = 0·0016); sublay mesh reinforcement vs primary suture an Odds Ratio of 0·55, (95% CI 0·30–1·00; *p* = 0·05). Seromas were found to be more frequent in patients after onlay mesh reinforcement (34 of 188) compared to primary suture (5 of 107; *p* = 0·002) or sublay mesh reinforcement (13 of 185; *p* = 0·002). The overall incidence of wound infection did not differ between treatment groups (14 of 107 primary suture; 25 of 188 onlay mesh reinforcement; and 19 of 185 sublay mesh reinforcement).

**Table 3:** Overview of 9 RCT's regarding prophylactic mesh in midline closure in high risk patients.

**Author**	**Year**	LoE*	**Type of surgery**	**Patients Mesh**	**Patients suture**	**SL/WL^†^**	**Mesh position**	**Type of mesh**	**Follow up month**
Gutierrez de la Pena	2003	2b	Gastro-intestinal	50	50	-	onlay	PP^‡^	36
Strzelczyk	2006	1b	Gastric bypass	36	38	-	sublay	PP	28
El-Khadrawy	2009	2b	Gastro-intestinal	20	20	4:1	pre-peritoneal	PP	36
Bevis	2010	1b	AAA	37	43	4:1	sublay	PP	25.4
Abo-Ryia	2013	2b	Gastric bypass	32	32	-	pre-peritoneal	PP	48
Caro-Tarrago	2014	1b	Colorectal	80	80	4:1	onlay	PP	13
Garcia-Urena	2015	1b	Colorectal	53	54	4:1	onlay	PP	24
Muysoms	2016	1b	AAA	56	58	4:1	sublay	PP	60
Jairam	2017	1b	AAA, BMI ≥ 27	373	107	4:1	188onlay185 sublay	PP	24

*LoE = Oxford Level of Evidence.

^†^Suture to Wound Length Ratio.

^‡^Polypropylene.

**Table 4:** Analysis of 8 RCT's of Meta analysis by Payne.

	**Primary Mesh Augmentation**		**Primary Suture**		**Risk Ratio**	
**Study/Year**	Incisional hernia	Total Number	Incisional hernia	Total Number	Weight in %	M-H, Random, 95% CI
Gutierrez de la Pena C 2003	0	44	5	44	6.3	0.09 (0.01, 2.32)
Strzelczyk 2006	0	36	8	38	6.5	0.06 (0.00, 1.04)
El-Khadrawy 2009	1	20	3	20	10.9	0.33 (0.04, 2.94)
Bevis 2010	5	37	16	43	63.4	0.36 (0.15, 0.90)
Abo-Ryia 2013	1	32	9	32	12.8	0.11 (0.01, 0.83)
Caro-Tarrago 2014	2	80	30	80	16.4	0.04 (0.01, 0.19)
Garcia-Urena 2015	6	53	17	54	27.8	0.28 (0.10, 0.77)
Muysoms 2016	0	56	16	58	5.2	0.02 (0.00, 0.39)
Total (95% CI)		360		367	100%	0.14 (0.07, 0.27)
Total events	15		104			

Heterogeneity: Tau^2^ = 0.15; Chi^2^ = 8.33, df = 7 (*p* = 0.30); I^2^ = 16%

Test for overall effect: Z = 5.80 (*p* < 0.00001)

## Technique

The initial step should include a precise median incision, with the linea alba sufficiently freed from subcutaneous fat, followed by dissection of the navel from the fascia, which is necessary in every case.

**Figure 1:**
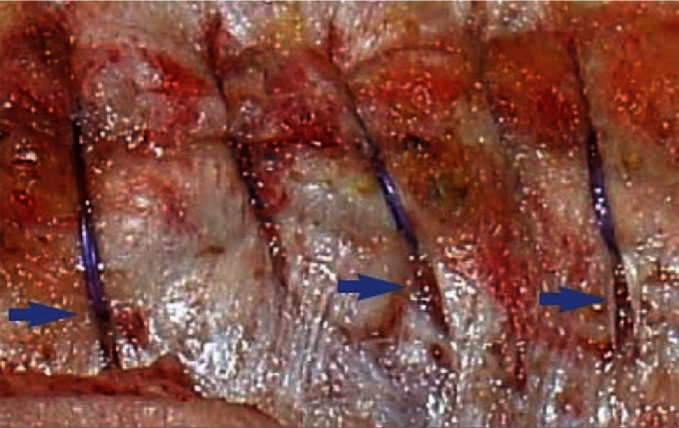
"Button holes" due to high tension aftermidline closure by large bite technique using a loop suture (blue arrows).

**Figure 2:**
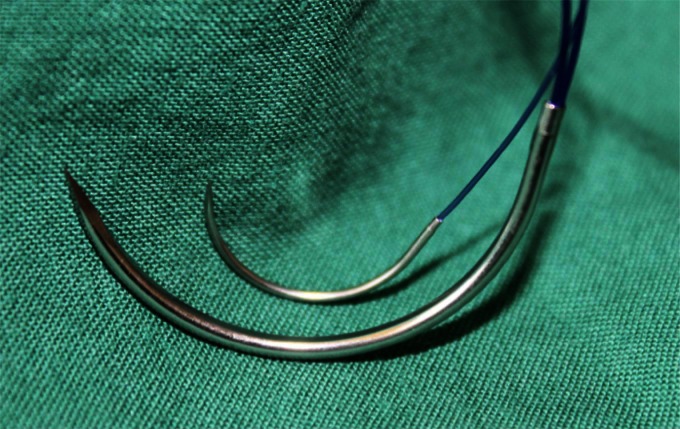
Comparison of loop suture (USP 1 with HR 48 needle) and single suture (USP 2/0 with HR 26 needle).

**Figure 3:**
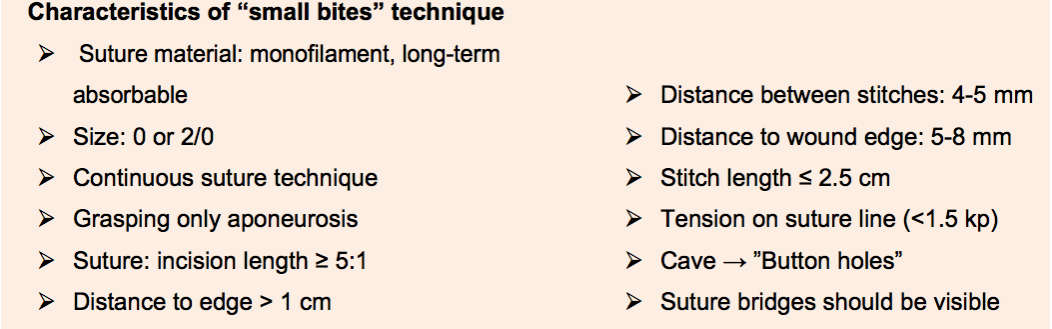
Characteristics of "small bites" technique.

**Figure 4:**
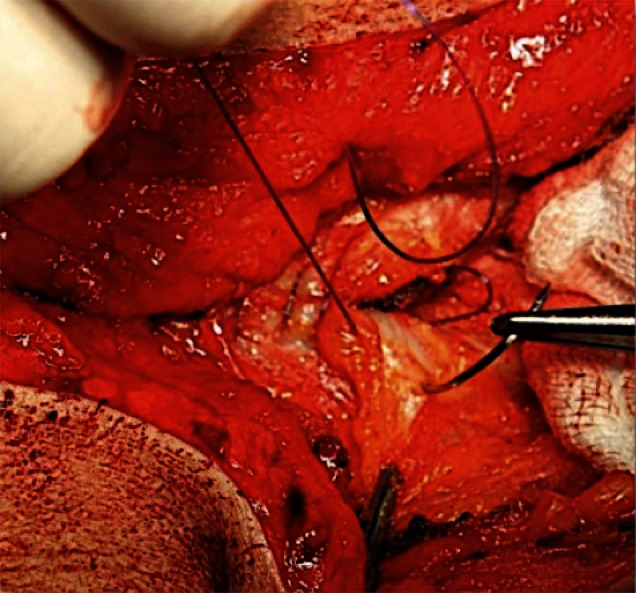
Small bites/stitch technique.

When closing, it is particularly important to ensure that the continuous suture tension is suitable for the type of tissue and the fascial edges, applying only a light tension on the tissue bridges in order to prevent the formation of “button-hole incisional hernias” (Figure 1). These criteria constitute the basis for a complication-free abdominal wall closure. Any excessive stress on the fascia or the linea alba during the intraoperative period beyond the “physiological extent” during the wakening and extubation phase, with the termination of muscular relaxation, inevitably leads to expansion of the stitch defect (Figure 2), which can subsequently manifest as a “button hole” or even the dreaded burst abdomen. Minimization of abdominal trauma is therefore the most crucial key in terms of improvement and development of surgical techniques. Based on biomechanical principles of abdominal wall tension the most important approach to minimize tissue trauma, is the distribution of suture tension over small tissue bridges by the use of appropriate needle size and suture strength (Figures 3, 4) (16). A further critical factor in preventing ‘button holes’, in addition to the suture technique, is the importance of a high elasticity of the suture material, which seems to play a major role in accordance to physiological studies of the abdominal wall.

 In an experimental study as well as in a controlled clinical randomized trial the “short stitch” technique proved by the Israelsson group (11, 16) the hypothesis of reduced tissue trauma and the positive impact on infection as well as scar hernia rates when using this technique. The significant superior results of this technique were based on a reduction of the distance between the stitches and the wound edge to between 5 and 8 millimeters and 5 millimeters from stitch to stich including only the aponeurosis leading to an increase of the suture/wound length ratio. The use of slowly absorbable sutures of size USP 2/0 (polydioxanone) and a half-circle round-bodied needle (thread length: 20 mm; arch length: 31 mm) in this suture technique resulted in a significant decrease of infection occurrence (5.2 versus 10.2%) as well as in the rate of incisional hernias (5.6 versus 18%) compared to the “long stitch” group after one-year follow up. Finally the study findings detected a twofold risk of wound infection and a fourfold risk of incisional hernia incidence in the “long stitch” group.

Another recently published multicenter randomized controlled study from the Netherlands, the STITCH trial, confirmed the significant advantages of small bites technique. The one-year results revealed an incisional hernia rate of 13% versus 21% with an odds ratio of 0·52 (95% CI 0, 31–0,87; *P* = 0,0131) in favor of the small bites technique (12).

Due to its compelling advantages, this concept was adopted in our department already five years ago. Despite the outstanding follow-up data available, the burst abdomen rate was the most important quality indicator: less than 0.5 percent. The initial concern of colleagues regarding sufficient strength has not been confirmed. After changing to the elastic suture material Monomax®, special attention was paid to ensuring moderate suture tension by means of the thread guide. Initial early postoperative burst abdomen complications were basically due to technical errors attributable to the learning curve. In our experience, the “short stitch” or “small bites” technique in fascia closures in abdominal wall repair with sublay mesh or onlay mesh augmentation can be used successfully. Furthermore, we were also able to perform secondary closure of the abdominal wall following open treatment of secondary peritonitis using an abdominal VAC system, with the described technique (17, 18). In these special situations protection and preservation of the median fascia is essential.

In the recently published ISSAAC study, the historical data of the INSECT study were compared to prospectively collected multicenter data (19). Different suture materials with different properties - PDS® or MonoPlus® from the INSECT study (10) - were compared to Monomax®, a new monofilament, ultra-long-term absorbable suture material with high elasticity and flexibility. The extra-long-term absorption time of this new suture material, together with its high elasticity, allows low-stress closure and long-term support of the healing process with minimal scar formation. Although this study compared to the INSECT study showed no significant differences regarding burst abdomen complications, wound infection and incisional hernias over a one-year period. The use of the monofilament suture evidenced a lower (*p* = 0.22) incisional hernia rate - 14 percent in the ISSAAC group of patients compared to 21.3 percent in the four centers participating in the INSECT study. Taking into account that in both studies the continuous suture technique with “large bites” was used, the findings of the Millbourn, Israelsson et al. study based on the “short bites” technique undoubtedly point to less surgical complications in the future.

In this regard, a multicenter, randomized trial, including centers located in Germany and Austria, the “ESTOIH” study was launched in 2015 comparing short stitch suture technique in combination with an elastic, extra-long term absorbable monofilament suture (Monomax®) with the long stitch suture technique (20). The primary endpoint is the incisional hernia rate 1 year postoperatively, assessed by ultrasound. The secondary parameters in this study will be the incidence of short term and long-term complications as well as costs, length of hospital stay and patients’ quality of life (EQ-5D-5 L). The follow up of patients after hospital discharge, will take place after 30 days and 1, 3, and 5 years after surgery. The study is still ongoing and the results are expected to be published in 2019.

The European Hernia Society published the guidelines on the closure of abdominal wall incisions by Muysoms et al (21) in 2015. The main recommendation of these guidelines is to use a non-midline approach to a laparotomy whenever possible to decrease the incidence of incisional hernias. The strong recommendation for the closure of elective midline incisions is to perform a continuous suturing technique and to avoid the use of rapidly absorbable sutures. In this review it is also suggested to use a slowly absorbable monofilament suture in a single layer aponeurotic closure technique without separate closure of the peritoneum. The current method of fascial closure in these guidelines is the recommendation to perform a small bite technique with a suture to wound length (SL/WL) ratio of at least 4:1.

These guidelines included the suggestion of additional prophylactic mesh augmentation, which was found to be effective and safe and suggested in high-risk patients, like aortic aneurysm surgery and obese patients.

The results of the newest study the “PRIMA”-trial (15) confirmed that the use of mesh reinforcement led to a significant reduction in the incidence of incisional hernia.

## Discussion

Taking into account all well known different risk factors concerning incisional hernia occurrence after elective midline closure, the evidence based on recently published randomized controlled trials has changed considerably. In 2010 the INLINE review was published by Diener et al (9) enrolling five systematic reviews and 14 trials in a total of 7,711 patients. The results of this analysis of available primary studies in the elective setting detected significant lower hernia rates using a continuous stitch technique in comparison to interrupted sutures (OR: 0.59; *p* = 0.001; Table 5) and slowly absorbable in comparison to rapid-absorbable suture material (OR: 0.65; *p* = 0.009). These finding were in contrast to the reviews published up to this time point. In the contrary the recent analysis of Henriksen et al (13) detected no significant differences on incisional hernia rate comparing non-absorbable and fast-absorbable sutures. But there seems to be a widely accepted consensus to close the midline after elective laparotomy as recommended by Diener et al (9), which is also found as a strong recommendation in the EHS-guidelines on the closure of abdominal wall incisions (21).

**Table 5:** Analysis of RCT's regarding comparison between continuous versus interrupted suture midline closure.

	**Suture technique Continous**		**Suture technique Interrupted**		**Odds Ratio**	
**StudyElective Procedure**	Incisional hernia	Total Number	Incisional hernia	Total Number	Weight	M-H, Random, 95% CI
Trimbos 1992	5	168	7	172	12.0%	0.72 (0.22, 2.32)
Brolin 1996	11	120	20	109	21.4%	0.45 (0.20, 0.99)
Colombo 1997	27	308	41	306	33.6%	0.62 (0.37, 1.04)
Seiler 2009	37	354	28	176	32.8%	0.62 (0.36, 1.05)
Total (95% CI)		950		763	100%	0.59 (0.43, 0.82)
Total events	80		68			

Heterogeneity: Tau^2^ = 0.00; Chi^2^ = 0.64, df = 3(*p* = 0.89); I^2^ = 0%

Test for overal effect: Z = 3.18 (*p* = 0.001)

The questions which technique to use for the continuous closure were answered based on the published data in experimental and clinical setting by the group of Israealsson (4, 5, 11, 16) in favor of the small stich technique. In 2015 the STITCH- trial revealed the significant advantages of small stitches and confirmed the results of Millbourne’s study (11), which was for a long time the only published RCT. Since these published randomized controlled trials the use of small stitch and small bites in a suture to wound length ratio of at least 5:1 in the elective midline closure should be rated as highly evidence based. In the recently published MATCH review (13) a total of 23 RCTs were included in the meta-analysis. The findings demonstrated that the use of slowly absorbable continuous sutures in small bites technique result in significantly less incisional hernias than a large bites technique in elective midline closure (OR 0.41; 95% CI 0.19, 0.86; Table 2).

Additional to this evidence the use of a prophylactic mesh in patients at risk in case of obesity (BMI <27) as well as in case of the presence of abdominal aortic aneurysm based on the results of the PRIMA – trial (15) seems convincing. These new evidences change the daily practice of closure techniques after elective midline laparotomies to a crucial extent. Thus up to now no RCT comparing the combination of small bites and prophylactic mesh has been published. The hypothesis of a significant improvement by this combination after midline laparotomy seems to be reasonable. The fact that prophylactic mesh implantation achieves better results in onlay in comparison to sublay position in the PRIMA-trial is of main importance for the feasibility of the mesh placement in midline laparotomy in general surgery, even for the group of non hernia specialized surgeons.

## Conclusions

In summary, the small bite/small stich-technique combined with prophylactic mesh implantation in high risk patients after midline laparotomies should be recommended to reduce significantly the rate of incisional hernias and other specific associated complications without a risk of the occurrence of negative side effects. Nevertheless we have to wait for the results of the ongoing studies for achieving any evidence of this new technique.

## Author Contributions

The author confirms being the sole contributor of this work and approved it for publication.

## Conflict of Interest Statement

The author declares that the research was conducted in the absence of any commercial or financial relationships that could be construed as a potential conflict of interest.

The handling Editor declared a past co-authorship with the author.
